# Gasdermin D Cleavage and Cytokine Release, Indicative of Pyroptotic Cell Death, Induced by Ophiobolin A in Breast Cancer Cell Lines

**DOI:** 10.3390/ijms27020618

**Published:** 2026-01-07

**Authors:** Santhalakshmi Ranganathan, Tolulope Ojo, Alagu Subramanian, Jenna Tobin, Alexander Kornienko, Angela Boari, Antonio Evidente, Mary Lauren Benton, Daniel Romo, Joseph H. Taube

**Affiliations:** 1 Department of Biology, Baylor University, Waco, TX 76706, USA; 2 Department of Chemistry and Biochemistry, Texas State University, San Marcos, TX 78666, USA; 3 Institute of Sciences and Food Production, National Research Council, 70126 Bari, Italy; 4 Institute of Biomolecular Sciences, National Research Council, 80078 Pozzuoli, Italy; 5 Department of Computer Science, Baylor University, Waco, TX 76706, USA; 6 Department of Chemistry and Biochemistry, Baylor University, Waco, TX 76706, USA

**Keywords:** breast cancer, ophiobolin A, necroptosis, PANoptosis, pyroptosis, cell death

## Abstract

An unmet challenge in managing breast cancer is treatment failure due to resistance to apoptosis-inducing chemotherapies. Thus, it is important to identify novel non-apoptotic therapeutic agents. Several non-apoptotic programmed cell death pathways utilize specific cellular signaling events to trigger lytic and pro-inflammatory cell death, examples of which are pyroptosis and necroptosis. Our study illustrates that ophiobolin A (OpA) is an anti-cancer agent that triggers lytic cell death in breast cancer cells, including triple-negative breast cancer (TNBC). This study reveals that OpA induces typical pyroptosis-like characteristics, including cellular swelling, plasma membrane rupture, GSDMD cleavage, and release of cytokines in breast cancer cells. However, the additional involvement of RIPK1 and induction of RIPK3 clustering in select cell lines suggest that multiple pathways may be triggered upon OpA treatment. The induction of pro-inflammatory cell death suggests potential applications for OpA in cancer treatment.

## 1. Introduction

Breast cancer is now the most prevalent form of malignant cancer, surpassing lung cancer, with approximately 2.3 million new cases diagnosed annually [1]. Despite considerable advancements in adjuvant therapy for breast cancer treatment, the widespread resistance to conventional pro-apoptotic treatments presents a significant challenge [2,3]. Resistance is associated with an elevated risk of recurrence and metastasis, resulting in high mortality rates [4]. There is a pressing demand for novel approaches to induce non-apoptotic programmed cell death (PCD) pathways to meet this clinical need.

Cell death is an essential event required in normal development and tissue homeostasis. The mechanism of PCD invoked by distinct drugs includes non-lytic (apoptosis, ferroptosis, paraptosis) and lytic forms (pyroptosis, necroptosis) and is highly relevant to anti-cancer treatment [5]. While apoptosis proceeds through caspase (Casp) cleavage, specifically Casp3, Casp6, and Casp7, and is initiated by Casp8 [6,7], necroptosis is accomplished in a caspase-independent manner and is triggered by a phosphorylation cascade involving receptor interacting protein kinase 1 (RIPK1), RIPK3, and mixed-lineage kinase domain-like (MLKL) protein [8]. In this pathway, phosphorylation of RIPK3 by RIPK1 triggers its kinase activity and subsequent phosphorylation of MLKL and disruption of plasma membrane integrity [8]. Characteristic features of other cell death pathways include iron overload and lipid peroxidation, triggering ferroptosis [9], and cytoplasmic vacuolation with dilation of the endoplasmic reticulum, triggering paraptosis [10].

While pyroptosis is similar to apoptosis in the involvement of caspases and chromatin condensation, unlike apoptosis, it exhibits cell swelling, membrane rupture, and the release of pro-inflammatory cytokines [11]. Membrane pore formation can be triggered through caspase-mediated cleavage of full-length Gasdermin D (GSDMD) into a 30 kDa fragment capable of polymerizing and perforating the cell membrane [12,13]. Given the complex features and interconnected signaling networks among these mechanisms, it is perhaps not surprising that features of apoptosis, necroptosis, and pyroptosis may be co-activated, termed PANoptosis [14]. In the context of breast cancer, the potential to leverage lytic cell death as a mechanism for therapy is promising, particularly in the context of resistance to apoptosis and for the capacity to synergize with immunotherapy approaches.

Ophiobolin A (OpA) is a sesterterpenoid produced by the pathogenic fungi of the *Bipolaris*, *Aspergillus*, and *Drechslera* genera that displays potent antiproliferative activity against multiple cancer cell lines, including chronic lymphocytic leukemia, breast cancer stem cells, and apoptosis-resistant glioblastoma [15,16,17,18]. However, both the mechanism of action and type of cell death pathway invoked by OpA have been difficult to establish and are likely cell type-dependent. OpA has been shown to induce paraptosis-like cell death, a form of non-apoptotic cell death, in human glioblastoma cells [19], necrosis in osteosarcoma cells [20], and autophagy in human melanoma cells [21]. We recently demonstrated that the triple-negative breast cancer (TNBC) cell line MDA-MD-231 is significantly more sensitive to the cytotoxic effect of OpA than the ER-positive breast cancer cell line MCF7 [22]; however, the mechanism of cell death that may underlie this difference was not established.

Breast cancer is a grouping of molecularly distinct diseases with distinct cells of origin and molecular markers. While patients with HER2-enriched and hormone receptor (HR)-positive breast cancers benefit from targeted treatments, patients with TNBC have more limited treatment options. In this study, we investigated the mechanisms of PCD induced by OpA treatment in multiple breast cancer cell lines representing specific molecular subtypes of the disease. Our study reveals that the mechanism of programmed cell death elicited by OpA is highly cell type-dependent. In a representative HER2-positive breast cancer cell line, OpA induces GSDMD cleavage and cytokine release, evidence for pyroptosis, while TNBC cell lines exhibit variable features of cell death pathways. These results illuminate the capacity of OpA to activate multiple features of lytic cell death against breast cancer cell lines. This is the first study showing Ophiobolin A to induce a combination of non-apoptotic PCD features in breast cancer.

## 2. Results

### 2.1. OpA-Induced Cytotoxicity Is Selective to Cancer Cells and Is Potent In Vivo

**Figure 1:**
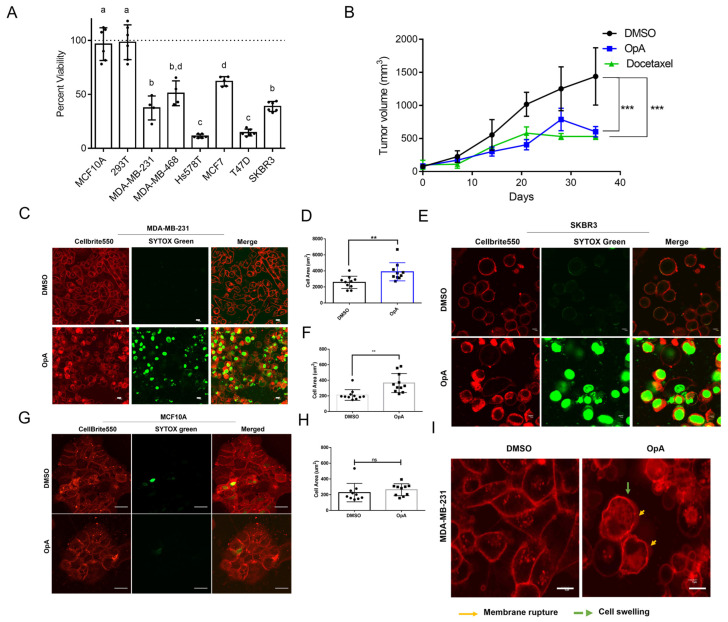
Ophiobolin A displays cancer cell-selective cytotoxicity via a lytic mechanism. (**A**) Cell viability of indicated cell lines treated with OpA at 500 nM for 24 h. The values presented are relative to the viability of vehicle-treated cells and normalized to 100%. Letters indicate statistically significant groupings via one-way ANOVA with Tukey’s correction for multiple hypothesis testing. (**B**) MDA-MB-231 cells were injected into the mammary fat pad of Scid/bg mice. Upon reaching 100 mm^3^, mice were treated with 5 mg/kg OpA or 5 mg/kg docetaxel i.p. weekly. Tumor growth curve assessed by caliper measurement. Tumor size data compared by two-way ANOVA with Sidak’s correction for multiple hypothesis testing. (**C**–**H**) Images and quantifications showing plasma membrane stain (Cellbrite550) and DNA stain (SYTOX green) in OpA (500 nM)-treated versus control cells for 6 h in indicated cell lines. Original magnification 20×; scale bar 10 µm. Cell size measured as area using ImageJ software (v1.54) and compared by Student’s *t*-test. (**I**) Confocal images of MDA-MB-231 cells treated with OpA (500 nM) showing membrane rupture and cell swelling compared to DMSO control. Original magnification 20×; scale bar 5 µm. All data are presented as the mean ± SD from at least three independent experiments. ** *p* < 0.01, *** *p* < 0.001, ns = not significant vs. control.

OpA has been demonstrated to possess cytotoxic activity towards various types of cancer cell lines, including human melanoma and glioblastoma [19,21]. In our previous work [22], we determined that epithelial–mesenchymal transition (EMT) enhances sensitivity to OpA and that breast cancer cell lines also exhibit differential sensitivity. We therefore sought to elucidate the molecular pathways involved in OpA-induced cytotoxicity across multiple breast cancer cell lines. We investigated the cytotoxicity of OpA in a panel of cell lines, which showed that OpA exhibits significant cytotoxicity in breast cancer cell lines but lacks activity towards non-tumorigenic human cells (Figure 1A). The results obtained from the analysis suggest that OpA is capable of selectively targeting cancer cells with low toxicity towards non-tumorigenic human breast cells (MCF10A) and human embryonic fibroblasts (293T). Corroborating these in vitro results, OpA, when dosed at 5 mg/kg, significantly reduces the tumor volume of MDA-MB-231 cells xenografted to immunocompromised mice, to a degree similar to docetaxel (Figure 1B). These findings suggest that OpA has the potential to serve as a treatment option for breast cancer patients; however, clarity as to the impact of OpA on cancer cells is needed.

We next evaluated the mechanism by which OpA induces cytotoxicity in breast cancer cell lines. To compare the cytotoxic effect of OpA with a known cytotoxic drug, MDA-MB-231 cells were treated with OpA alone or in combination with docetaxel. Our results demonstrate that a combination of OpA and docetaxel displays a greater cytotoxic effect than each agent alone, indicating a potential complementary mode of action (Figure S1A). In glioblastoma and melanoma, OpA has been shown to induce paraptosis and autophagy [19,21]. To test for paraptosis induction, we examined the capacity of spautin-1, an inhibitor of USP10 that is required for paraptosis [23], to rescue OpA-induced cytotoxicity. Our findings reveal that spautin-1 was ineffective in rescuing OpA-induced cytotoxicity against MDA-MB-231 cells and MCF7 cells (Figure S1B), indicating a lack of dependency on USP10. We next tested for evidence of the induction of autophagy through the detection of autophagolysosomes. While rapamycin was sufficient to cause an increase in autophagolysosome staining in MDA-MB-231 cells, OpA failed to do so (Figure S1C), supporting the idea that autophagy is not induced.

We next evaluated the role of the caspase-dependent apoptotic cell death pathway in OpA-induced cytotoxicity by employing a pan-caspase inhibitor, zVAD. zVAD partially rescued caspase-dependent cell death in two of three TNBC cell lines (MDA-MB-231 and MDA-MB-468, but not Hs578T) (Figure S2A), indicating that OpA-induced cell death involves caspase activation. However, the magnitude of rescue by zVAD was minimal, suggesting that OpA may induce cell death through additional pathways. Additionally, OpA-treated MDA-MB-231 cells show no signs of membrane blebbing or apoptotic bodies as observed in the staurosporine-treated cells (Figure S2B).

### 2.2. Ophiobolin A Triggers Morphological Changes Consistent with Lytic Cell Death

To obtain morphological evidence of the cell death mechanism employed by OpA, we checked for structural changes in the plasma and nuclear membranes upon exposure to OpA by visualizing the plasma membrane and DNA. Both MDA-MB-231 (Figure 1C) and SKBR3 (Figure 1E) cell lines exhibited ruptured membranes and took up Sytox Green, thus suggesting compromised membrane integrity consequent to OpA treatment. To assess the effect of OpA on non-transformed cells, MCF10A cells were treated with OpA and stained similarly. The results show that MCF10A cells are not affected by OpA treatment, as indicated by the intact membrane integrity (Figure 1G). Moreover, cells that respond to OpA treatment exhibit a noticeably swollen appearance, as evidenced by the quantification of the cell area of MDA-MB-231 (Figure 1D) and SKBR3 cells (Figure 1F) but not MCF10A cells (Figure 1H). This suggests that the cell-targeting ability of OpA is selective to cancer cells and does not affect non-cancer cells. Rupture of the plasma membrane is characteristic of the induction of lytic cell death [24]. Studies have documented the occurrence of plasma membrane rupture and cell swelling during both necroptosis and pyroptosis, two forms of PCD [24]. Thus, we next sought to measure the capacity of OpA to activate features of these pathways.

### 2.3. Ophiobolin A Requires RIPK1 Activity and Can Induce Necrosome Formation upon RIPK3 Overexpression

**Figure 2:**
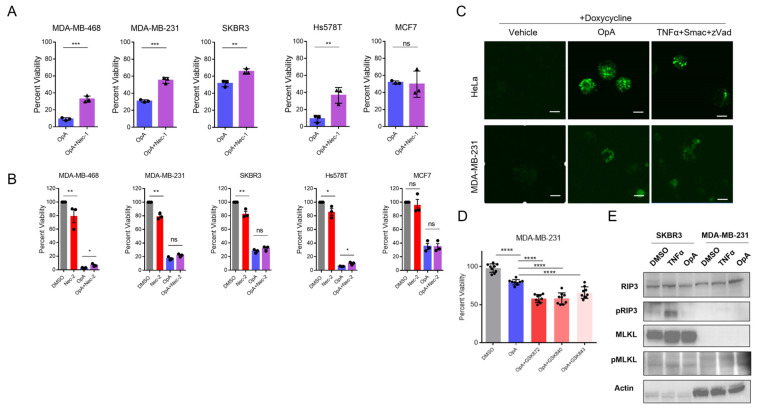
Ophiobolin A activity is dependent on RIPK1 but not RIPK3. (**A**) Viability of cells treated with 500 nM OpA in presence or absence of necrostatin-1 (100 µM). OpA+Nec-1 compared to OpA by Student’s *t*-test. (**B**) Viability of cells treated with 500 nM OpA in presence or absence of necrostatin-2 (10 µM). The values presented are relative to the viability of vehicle-treated cells and normalized to 100%. Statistical significance of select pairs highlighted following 1-way ANOVA. (**C**) Confocal images of transiently transfected RIPK3-GFP MDA-MB-231 cells and stably transfected RIPK3-GFP HeLa cells treated with doxycycline (20 μg/mL) for 24 h and with 500 nM OpA or 20 ng/mL TNFα+100 nM Smac/LCL161+20 μM zVad or vehicle for 24 h for visualization of the necrosome. Original magnification 20×; scale bar 20 µm. (**D**) Viability of OpA-treated cells in presence or absence of RIPK3 inhibitors (GSK872, GSK840, GSK843). The values presented are relative to the viability of DMSO-treated cells and normalized to 100%. All groups compared via one-way ANOVA with Tukey’s correction for multiple hypothesis testing. (**E**) Immunoblot analysis of total and phospho-RIPK3, total and phospho-MLKL using lysates of OpA- or TSZ-treated cells. β-Actin was used as an internal control. Cells were treated for 24 h. Viability data are presented as the mean ± S.E.M. from at least three independent experiments. * *p*< 0.05, ** *p* < 0.01, *** *p* < 0.001, **** *p* < 0.0001, ns = not significant.

Upon observing signs of lytic cell death, we opted to investigate necroptotic cell death (programmed necrosis), which acts through receptor-interacting serine/threonine-protein kinase 1 (RIPK1). We tested whether Necrostatin-1 (Nec-1), an RIPK1 inhibitor, was sufficient to rescue OpA-induced cytotoxicity. Our results demonstrate that Nec-1 blocks cell death in all the cell lines we evaluated, except for MCF7 (Figure 2A). While Nec-1 has been shown to inhibit RIPK1 activity, some off-target effects have been demonstrated, particularly for IDO [25]. To examine the specific contribution of RIPK1 to OpA-mediated cell death, we tested Necrostatin 2 racemate (Nec-2) for its capacity to rescue OpA-induced cytotoxicity [26]. To our surprise, we observed moderate cytotoxicity from Nec-2, even at 10 µM (Figure 2B). This cytotoxicity was not observed in combination with OpA, for which 2 cell lines (MDA-MB-468 and Hs578T) exhibited a statistically significant rescue in cytotoxicity and two cell lines with Nec-2 induced cytotoxicity (MDA-MB-231 and SKBR3) observed no statistically significant difference (Figure 2B). MCF7 cells were not similarly affected by Nec-2 and showed no rescue of OpA (Figure 2B). Nevertheless, the magnitude of rescue effect of Nec-2 is minimal in comparison to Nec-1, indicative of a minimal or absent role for RIPK1 in OpA-driven cytotoxicity.

RIPK1 is known to phosphorylate a downstream kinase, receptor-interacting serine/threonine-protein kinase 3 (RIPK3), which then interacts in a protein complex with a mixed-lineage kinase domain like pseudokinase (MLKL) in a structure termed the necrosome, which is a crucial step in inducing necroptotic cell death [27]. The RIPK3 mRNA is typically expressed at lower levels in breast cancer cells when compared to normal cells, as per the TCGA database, and can be silenced by DNA methylation [28]. Thus, to further examine the capacity of OpA to activate this pathway, and, in particular, induce necrosomes, we overexpressed GFP-tagged RIPK3 in MDA-MB-231 cells and in HeLa cells, a model previously published [27]. The formation of puncta indicative of necrosomes was visualized via fluorescence microscopy. In contrast to vehicle control, OpA caused both MDA-MB-231 cells and HeLa cells expressing RIPK3-GFP to exhibit fluorescent puncta, similar to the formation of necrosomes induced by the positive control cocktail, TNFα+Smac+zVad (TSZ) [27,29] (Figure 2C). However, MCF-7 cells overexpressing RIPK3 fail to exhibit puncta upon OpA treatment (Figure S3A). Additionally, OpA induces intra-cellular calcium accumulation, a potential trigger of necroptosis, as noted in other studies [30] (Figure S3B). These data indicate that OpA relies upon RIPK1 for cytotoxic activity and has the capacity to induce necrosome formation in cancer cells upon the overexpression of RIPK3.

To provide additional insights on the role of RIPK3 in OpA-induced cytotoxicity, we tested the effect of the RIPK3 inhibitors GSK840, GSK843, and GSK870. However, all three inhibitors were unable to rescue cell death (Figure 2D), indicating that endogenous RIPK3 may not be active in inducing necroptosis in response to OpA. To ascertain whether the kinase cascade characteristic of necroptosis is activated by OpA, we analyzed the phosphorylation of endogenous RIPK3 and MLKL proteins, using Western blotting. Our data indicate that OpA was insufficient to induce the phosphorylation of RIPK3 and MLKL proteins in either MDA-MB-231 or SKBR3 cells (Figure 2E), despite the capacity of TSZ to phosphorylate RIKP3 in SKBR3 cells. These data highlight an intriguing potential for OpA to trigger necrosome formation despite a minimal role for RIPK1 activity in OpA-induced cytotoxicity.

### 2.4. Ophiobolin A Enhances the Expression of Cytokines and Triggers Inflammatory Genes

**Figure 3:**
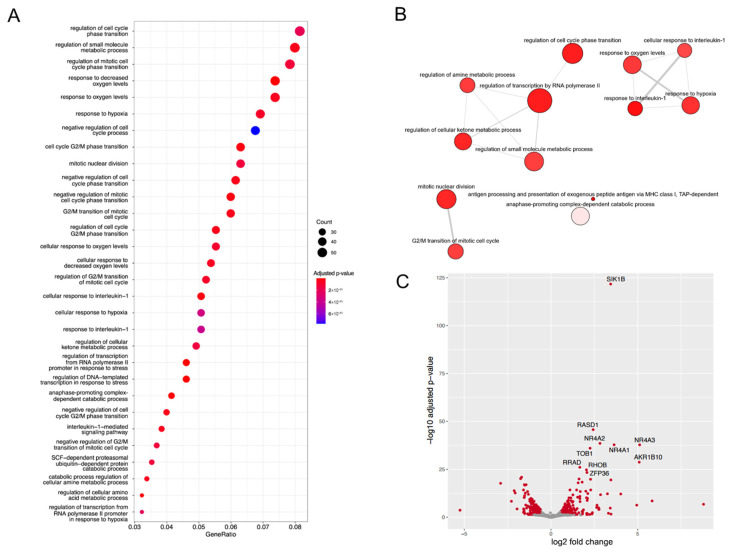
Ophiobolin A induces a gene expression signature indicative of response to cytokines. (**A**) Gene ontology (GO) analysis of differentially expressed genes from MDA-MB-231 cells treated with 250 nM OpA for 3 h. (**B**) GO categories with *p*-values < 10^−10^ were analyzed via Revigo to highlight non-redundant pathways. Node size indicates the frequency of the GO term in the ontology, while node color indicates the *p*-value from the GO enrichment. The edges link similar GO terms (above a preset threshold), with thicker edges between terms with a higher degree of similarity. (**C**) Volcano plot showing differentially expressed genes from OpA-treated MDA-MB-231 cells.

In addition to necroptosis, lytic cell death may be accomplished through pyroptosis, which involves up-regulation and release of pro-inflammatory cytokines. Gene ontology (GO) analysis changes in mRNA expression upon OpA treatment of MDA-MB-231 cells show elevated pathways including genes involved in cell cycle regulation and metabolism, as well as the response to interleukin-1 (Figure 3A,B). Additionally, NR4A1-NR4A3 (Nur77, Nurr1, and Nor-1), which are immediate early genes induced by growth factors, cytokines, inflammatory and physiological stimuli, and cellular stress [31], are highly activated in OpA-treated MDA-MB-231 cells (Figure 3C), suggesting that OpA might activate pyroptosis.

**Figure 4:**
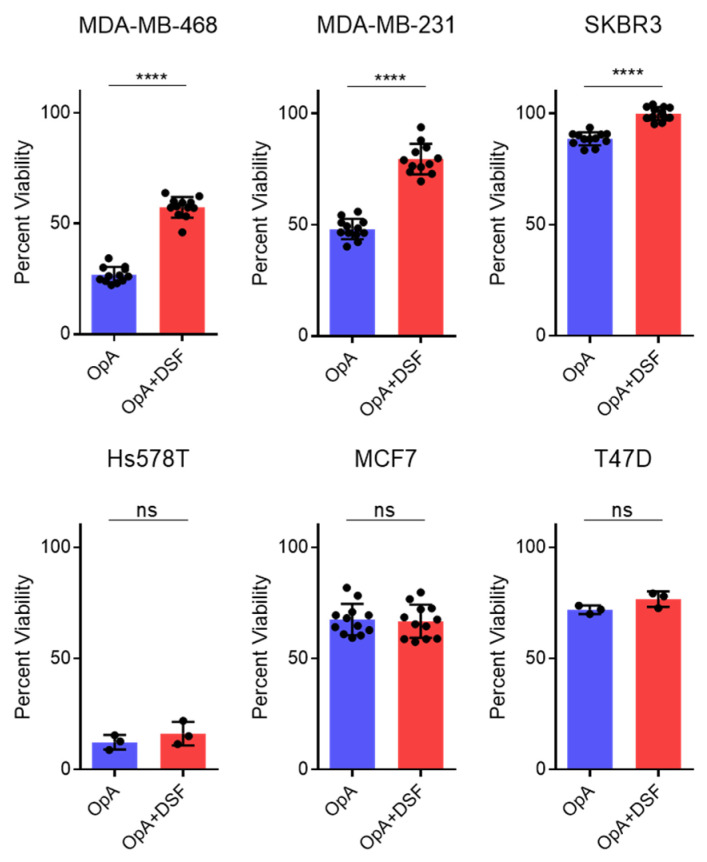
Ophiobolin A cytotoxic activity is blocked by disulfiram. Viability of OpA-treated cells in presence or absence of disulfiram (1.25 µM) for 24 h. All data are presented as the mean ± S.E.M. from at least three independent experiments. Significance calculated using Student’s *t*-test. **** *p* < 0.0001, ns = not significant.

To further test this hypothesis, we examined the ability of disulfiram, an inhibitor of Gasdermin D (GSDMD) cleavage and pyroptotic cell death [32,33], to rescue OpA-induced cytotoxicity. Our data demonstrate that disulfiram exhibits a notable capacity to mitigate the cytotoxic effects induced by OpA in two of the three TNBC cell lines examined, although this effect was not observed in all cell lines (Figure 4).

**Figure 5:**
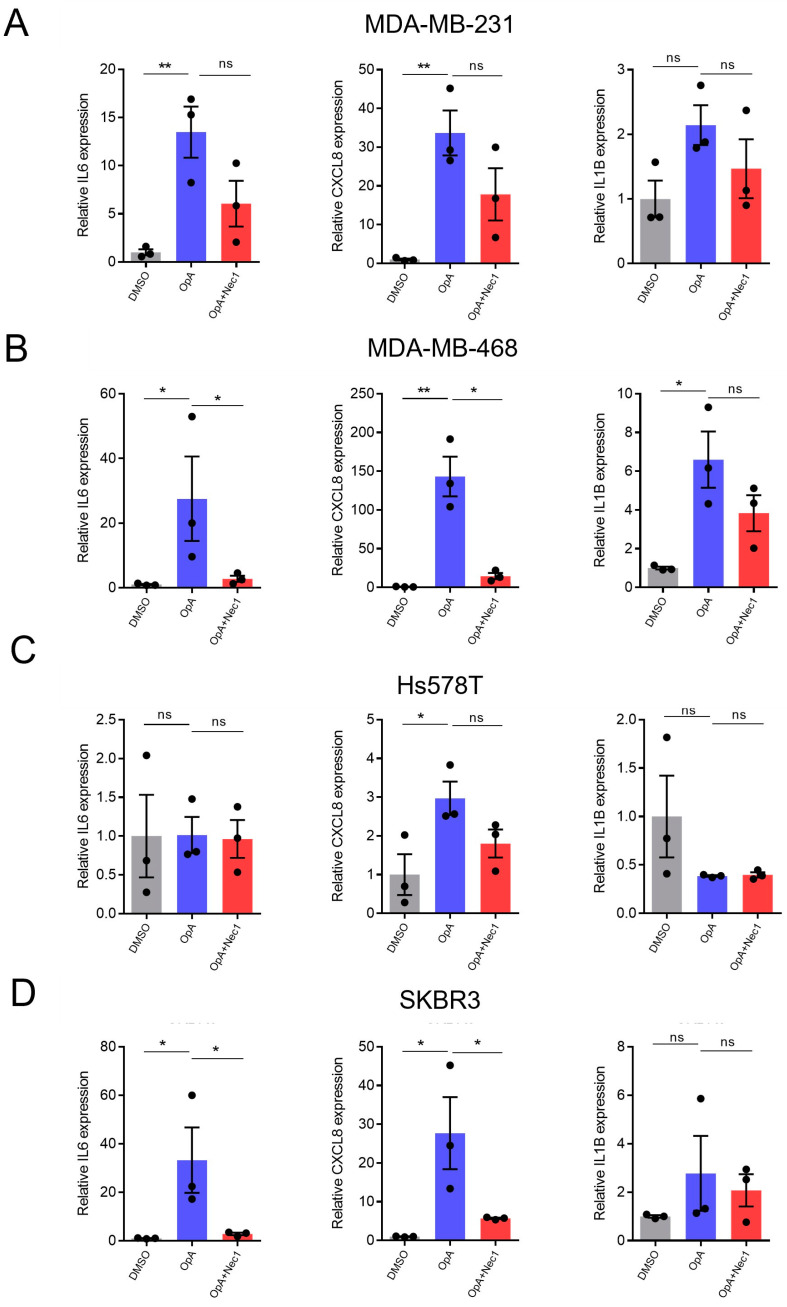
Ophiobolin A induces cytokine expression in breast cancer cell lines in a subtype-specific manner that is partially blocked by Nec-1. Real-time quantitative PCR analysis of the indicated mRNAs was performed for MDA-MB-231 (**A**), MDA-MB-468 (**B**), Hs578T (**C**), and SKBR3 (**D**) cells upon treatment with OpA, Nec-1, or vehicle control. All data are presented as the mean ± S.E.M. from at least three independent experiments. Significance calculated using one-way ANOVA with Dunnet’s correction for multiple hypothesis testing. * *p* < 0.05, ** *p* < 0.01, ns = not significant.

As upregulation of cytokine expression and secretion are key features of pyroptosis [34], we assessed the levels of three key cytokines: interleukin-6 (*IL6*), interleukin-8 (*CXCL8*), and interleukin-1 beta (*IL1B*). Our results reveal that OpA significantly elevates mRNA levels of inflammatory cytokines *IL6*, *CXCL8*, and *IL1B* in MDA-MB-231 cells (Figure 5A), *IL6*, *CXCL8*, and *IL1B* in MDA-MB-468 cells (Figure 5B), *CXCL8* in Hs578T cells (Figure 5C), and *IL6* and *CXCL8* in SKBR3 cells (Figure 5D), suggesting the induction of pyroptosis by OpA. Given our earlier observation that Nec-1 suppresses OpA-induced cell death in these four cell lines (Figure 2), we further asked if Nec-1 suppresses the induction of these cytokines. In MDA-MB-468 and SKBR3 cells, Nec-1 suppressed the induction of *IL6* and *CXCL8* (Figure 5B,D), while the results in MDA-MB-231 cells were not statistically significant.

### 2.5. Ophiobolin A Facilitates Release of Cytokines and Cleavage of Gasdermin D Favoring Pyroptotic Cell Death

**Figure 6:**
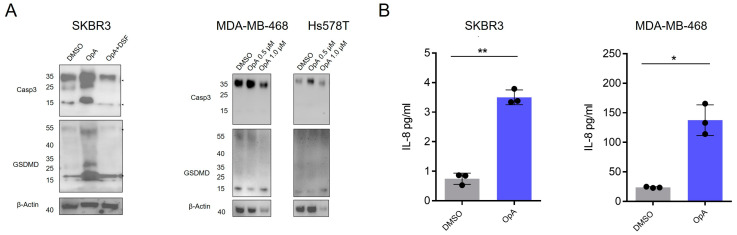
Ophiobolin A facilitates Gasdermin D cleavage and the release of IL-8. (**A**) Immunoblots showing the expression or cleavage of caspase 3 and GSDMD upon treatment with 500 nM OpA in the presence or absence of disulfiram for 12 h. (**B**) Levels of IL-8 protein released in conditioned media of indicated cell lines treated with OpA with or without disulfiram as in (**A**), measured using ELISA. All data are presented as the mean ± S.E.M. from at least three independent experiments. Significance calculated using one-way ANOVA with Dunnet’s correction for multiple hypothesis testing. * *p* < 0.05, ** *p* < 0.01.

The release of pro-inflammatory cytokines IL-8 and IL-1β has been attributed to a mechanism of pore formation in plasma membrane dependent on GSDMD [35]. Cleavage of GSDMD and caspase-3 are well-established markers of pyroptotic cell death and necessary for membrane pore formation [36,37,38]. Our Western blot analysis reveals evidence of GSDMD cleavage in SKBR3 cells (Figure 6A), suggesting that OpA is capable of inducing pyroptotic cell death in HER2-positive cells. However, TNBC cell lines do not demonstrate GSDMD cleavage (Figure 6A) upon OpA treatment. We also observed enhanced cleavage of caspase-3 upon OpA treatment of SKBR3 cells, which was suppressed by DSF (Figure 6A). Caspase-3 is known to be activated in several cell death pathways [39] but can inhibit the pore formation activity of GSDMD by cleaving it into a 43 kD fragment [40]. Notably, the GSDMD fragment (30 kDa) observed in our Western blot analysis is consistent with cleavage via an activation pathway rather than the product of caspase-3 [41]. To test for the release of mature cytokines, we measured the protein concentration in conditioned media of cells treated with OpA. OpA significantly induces IL-8 release when compared to control in SKBR3 and MDA-MB-468 cells (Figure 6B). Altogether, our data indicate that, in SKBR3 cells, OpA induces transcription of pro-inflammatory cytokines, GSDMD cleavage, and cytokine release, while in TNBC cells, cytokine upregulation and release can occur in the absence of GSDMD cleavage.

## 3. Discussion

The ability of tumor cells to evade apoptosis is a critical factor in the development of cancer [42]. Despite advancements in the management of breast cancer, the prognosis for patients with advanced disease remains unfavorable, mainly due to multidrug resistance (MDR) to cytotoxic chemotherapy drugs [2,3]. Therefore, identifying and characterizing additional therapeutic agents is critical in the fight against breast cancer.

Our research has demonstrated that OpA triggers the formation of membrane pores and the uptake of Sytox Green, indicating the induction of lytic cell death in breast cancer cells, including activation of GSDMD in HER2-positive SKBR3 cells or via an undetermined mechanism in TNBC cells. While OpA has been previously shown to induce paraptosis and autophagy in glioblastoma and melanoma cells [19,21], our study did not reveal significant induction of paraptosis or autophagy in breast cancer cells. However, we did find that zVAD, a pan-caspase inhibitor, rescues OpA-induced cell death in breast cancer cells despite the lack of annexin-V positivity. Therefore, our results suggest that OpA-induced cell death is mediated by caspase-dependent mechanisms in breast cancer cells but does not necessarily involve the apoptotic pathway.

Necroptosis and pyroptosis are two forms of programmed cell death that belong to the lytic pathway, characterized by the rupture of the cell membrane [43]. Necroptosis, as its name suggests, is a programed form of necrosis, which functions through the activation of specific signaling pathways [44]. RIPK1 is thought to be a crucial checkpoint regulator of apoptosis and necroptosis [45]. RIPK1/RIPK3 necrosomes phosphorylate MLKL, leading to the formation of membrane pores that promote necroptosis in the absence of caspase activation [14,46]. In our study, RIPK1 inhibitors significantly blocked OpA-induced cell death in breast cancer cells, including TNBC, indicating that RIPK1 is involved in triggering the cell death pathway. Considering that RIPK3 is suppressed in tumor cells compared to normal cells, our RIPK3-overexpressing cells showed distinct puncta (necrosome) formation on OpA treatment, similar to the puncta shown by Sun et al. [27]. However, OpA-treated cells failed to show phosphorylation of RIPK3 or MLKL in breast cancer cell lines.

While both pyroptosis and necroptosis are lytic cell death pathways with inflammatory characteristics, such as the release of damage-associated molecular patterns (DAMPs), they differ in their activation sequence. Pyroptosis can be a response to the presence of pathogen-associated molecular patterns (PAMPs) or DAMPs triggered by caspase-1 activation, resulting in the activation of cytokines IL-1β and IL-18 [6]. In contrast, necroptosis is a secondary defense mechanism triggered by RIPK3 activation in response to the inhibition of pro-apoptotic caspase-8 [7]. The two pathways are related in that DAMPs released during necroptosis can activate pyroptosis through the regulation of the inflammatory axis, most notably, IL-1α [5,8,9]. Pyroptosis has been shown to be mediated by the cleavage of multiple gasdermin family members and the expression and release of inflammatory caspases IL-1β, IL-4, IL-5, and IL-11 [47,48]. Previous research has demonstrated that cleavage of GSDMD results in the release of its N-terminal domain, leading to the formation of pores in the cell membrane, cell swelling, rupture, and death [12]. In our study, we observed cleavage of GSDMD in SKBR3 cells, indicating that this event may be responsible for the formation of membrane pores and cell swelling. Additionally, inhibition of GSDMD cleavage by disulfiram [32] (Figure 6A) suppressed caspase-3 cleavage, indicating that pore formation may be critical for subsequent caspase activation. This aligns with the recent finding by Chan et al. (2025), who demonstrated that caspase-mediated GSDMD cleavage is essential for pyroptotic cell death in macrophages and may occur even in the absence of canonical inflammasome activation [49]. OpA treatment also resulted in elevated transcription of cytokines IL-6, IL-8, and IL-1β and a release of IL-8, as observed in studies of pyroptosis [35]. Furthermore, GO analysis of RNA-seq data revealed an elevated interleukin-1-mediated signaling pathway.

Our study reveals that OpA induces typical pyroptotic characteristics, including cell membrane rupture, cell swelling, the release of cytokines, and GSDMD cleavage in select breast cancer cells. However, our research also raises the question of how RIPK1 is involved in OpA-induced pyroptosis. The potential for coordinated action of RIPK1 and GSDMD points to a novel axis linking inflammatory signaling to membrane permeabilization. In line with previous observations [50], we speculate that there could be a crosstalk between necroptosis and pyroptosis in inducing cell death in breast cancer cells. Such crosstalk between cell death pathways has been recently reported in other studies, where apoptosis, necroptosis, and pyroptosis interact to activate inflammatory cell death, in a process termed PANoptosis [14,50,51,52,53]. In PANoptosis, despite RIPK3-MLKL hyperactivation, RIPK1 efficiently drives inflammasome activation and IL-18 secretion [50]. Consistent with this, a recent report highlighted that RIPK1-dependent caspase-8 activation leads to GSDMD cleavage and pyroptosis in response to *Yersinia* infection and that alternative pathways including caspase-11 are recruited when RIPK1 or caspase-8 is impaired [49]. This highlights the plasticity and interplay among cell death pathways and their markers.

## 4. Materials and Methods

### 4.1. Cell Culture and Stable Cell Line

Human breast cancer cell lines MCF7 (RRID: CVCL_0031), MDA-MB-231 (RRID: CVCL_0062), MDA-MB-468 (RRID: CVCL_0419), Hs578T (RRID: CVCL_0332), and SKBR3 (RRID: CVCL_0033), as well as the non-transformed mammary cell line MCF10A (RRID: CVCL_0598) and fibroblast cell line 293T, were purchased and cultured as recommended by American Type Culture Collection (ATCC, Manassas, VA, USA). All the cell lines were cultured with respective media (Gibco, Fisher Scientific, Hampton, NH, USA) containing 10% Fetal Bovine Serum (Gibco, USA) or 5% horse serum (Cytiva, HyClone, Logan, UT, USA) and 1% penicillin and streptomycin in a humidified incubator at 37 °C and 5% CO_2_. All cell lines were routinely tested for the absence of mycoplasma contamination and for identity by STR profiling (UT MDACC Cytogenetics and Cell Authentication Core, Houston, TX, USA). HeLa cells and plasmid expressing RIPK3 were a kind gift from Dr. Zhigao Wang [27].

### 4.2. Ophiobolin A Source and Purification

OpA was produced by *Drechslera gigantea* and purified from the fungal culture filtrates as reported previously [54]. Briefly, the fungus was grown and maintained on Petri dishes containing PDA (Oxoid, Fischer Scientific, Basingstoke, UK). The culture filtrates were lyophilized, redissolved in distilled water, and extracted with EtOAc. The combined organic extracts were dehydrated, filtered, and evaporated under reduced pressure. The brown oily residue was fractionated by column chromatography, and crude OpA was crystallized as white needles.

### 4.3. Cell Culture Treatments and Viability Assay

To assess the cell viability, cells were seeded at the density of 5000 cells per well in 96-well plates. Compounds used include zVAD (Cat#S7023, Selleckchem, Houston, TX, USA), Ferrostatin-1 (Cat#SML-0583, MilliporeSigma, St. Louis, MO, USA), Spautin-1 (Cat#56756910MG, MilliporeSigma), Disulfiram (Cat#HY-B024, Tocris Bioscience, Bristol, UK), Necrostatin-1 (Cat# J65341FPL, ThermoScientific, Waltham, MA, USA), Necrostatin-2 racemate (T7504, TargetMol, Boston, MA, USA), GSK872 (Selleckchem), GSK840 (Selleckchem), and GSK843 (Selleckchem). Cells were treated with OpA in the presence or absence of the inhibitors for 24 h; later, Cell Titer Blue (CTB) (G8081, Promega, Madison, WI, USA) was added directly to each well according to the manufacturer protocol, wells were incubated for 3 h at 37 °C, and the fluorescent signal was recorded at 560_Ex_/590_Em_. Cells treated with a combination of TNF-α (MilliporeSigma Cat# GF023), Smac/LCL161 (CAS#1005342-46-0, MedChemExpress, Monmouth Junction, NJ, USA), and zVAD were used as positive controls for necroptosis.

### 4.4. Autophagy Assay

Cells were cultured to 85% confluence, dosed, washed with PBS, and stained with CYTO-ID Green Detection Reagent according to the manufacturer’s instructions (Cat# 1750200, Enzo Life Sciences, Farmingdale, NY, USA). The individual cellular fluorescence was measured using the Lionheart FX Automated Microscope (BioTek, Santa Clara, CA, USA) under the cellular analysis protocol. The fluorescence captured was then quantified and analyzed using GraphPad Prism.

### 4.5. Annexin V Assay

For evaluation of apoptosis, cells were labeled using the Annexin V-FITC early apoptosis detection kit (Cat#640914, Biolegend, San Diego, CA, USA) following the manufacturer’s protocol. Briefly, cells were plated on 10 cm dishes and treated with or without OpA or staurosporine (1 µM) for 24 h. Following trypsinization, the harvested cells were pelleted and resuspended in Annexin V FITC in binding buffer and incubated for 30 min at room temperature in the dark, and then propidium bromide (PI) was added for FACS analysis using BeckmanFACS Melody (BD Biosciences, Franklin Lakes, NJ, USA).

### 4.6. Intracellular Calcium Accumulation Assay

Cells were seeded in a microplate and treated with varying concentrations of OpA for 24 h. Following treatment, the medium was replaced with fresh culture medium containing 5 µM Fluo-3 AM (F1241, Thermo Fisher Scientific), and cells were incubated for 30 min at 37 °C to allow dye loading. The cells were then washed three times with Hank’s Balanced Salt Solution (Fisher Scientific) to remove excess dye. Fluorescence images were acquired using an excitation wavelength of 488 nm on an epi-fluorescent microscope (Ts2R-FL, Nikon, Melville, NY, USA).

### 4.7. Live Cell Imaging

To interrogate the formation of necrosomes, RIPK3-overexpressing cells were seeded in 96-well glass-bottom plates (Ref4580, Corning, Mediatech Inc., Manassas, VA, USA), treated with DMSO or OpA or TNF-α (YD370435, Thermo Scientific) and zVAD along with doxycycline (Cat# BP26535, Fisher Scientific) for 24 h. To examine the integrity of the plasma membrane, the cells were treated with or without OpA for 4 h and stained with Cellbrite550 (Cat#30105A, Biotium, Fremont, CA, USA) and SYTOX green (S7020, Fisher Scientific) in respective media for 30 min at 37 °C. Later, the cells were imaged using a confocal microscope. Cells treated with Thapsigargin (Cat#586005, SigmaMillipore) were used as positive controls for necroptosis.

### 4.8. Immunoblotting

Cells were lysed with RIPA buffer (Thermo Scientific Cat#J63306.AP) supplemented with 1X protease inhibitor (Thermo Scientific Cat#78430) and 1X phosphatase inhibitor (Thermo Scientific Cat#78428). The protein concentrations were determined using a BCA reagent kit (Thermo Scientific Cat#23225) and then an equal amount (50 µg) of whole cell lysates were subjected to 4–12% SDS-PAGE, transferred onto a 0.45 µm PVDF membrane (Thermo Scientific Cat#.88520). The membranes were incubated with appropriate primary antibodies overnight. The primary antibodies used were as follows: RIP3 (13526S, Cell Signaling Technology, Danvers, MA, USA), pRIP3 (93654S, Cell Signaling), MLKL (66675-14g, Proteintech, Rosemont, IL, USA), pMLKL (ab187091, Abcam, Cambridge, UK), Caspase3 (9662S, Cell Signaling Technology), Caspase1 (4199, Cell Signaling Technology), Gasdermin D (ab215203, Abcam), Actin (612657, BD Bioscience), HRP-conjugated anti-mouse secondary antibody (7076S, Cell Signaling Technology), and anti-rabbit secondary antibody (7074S, Cell Signaling Technology). Immunoblots were treated with ECL Prime (Cytiva) and imaged using the Biorad ChemiDoc Imaging system. β-Actin was used as an internal standard. All primary antibodies were diluted 1:1000 with Tris-buffered saline Tween. Beta-actin and secondary antibodies were diluted 1:2000. Primary antibodies were incubated overnight at 4 °C, and secondary antibodies were incubated for 2 h at room temperature.

### 4.9. Reverse Transcription and Real-Time Quantitative PCR

Total RNA was isolated from cells treated with or without OpA (1 μM) for 6 h, using TRIzol reagent (15596018, Ambion, Fisher Scientific) according to the manufacturer’s protocol. cDNA was generated using 250 ng of RNA with MultiScribe reverse transcriptase (4319983, Fisher Scientific) according to the manufacturer’s protocol. The synthesized cDNA was used to perform real-time quantitative PCR using qPCR blue mix (AZ2305, Azura Genomics, Raynham, MA, USA) by Quantstudio5 system (Applied Biosciences, Fisher Scientific). The comparative Ct method was used for relative mRNA quantification using the formula 2^−∆∆Ct^ [55]. All reactions were performed as at least three independent experiments. All primers were obtained from Integrated DNA Technologies (Newark, NJ, USA). Primers were added to a final concentration of 2.3 μM. Primer sequences were as follows: IL6, forward-TGAGGAGACTTGCCTGGTGA, reverse-CTGCACAGCTCTGGCTTGTT, human CXCL8, forward-ATGACTTCCAAGCTGGCCGT, reverse-TCCTTGGCAAAACTGCACCT, IL1B, forward-GCAAGGGCTTCAGGCAGGCCGCG, reverse-GGTCATTCTCCTGGAAGGTCTGTGGGC human ACTB, forward-CATGTACGTTGCTATCCAGGC, reverse-CTCCTTAATGTCACGCACGAT. All primer amplifications were carried out in thermocycling conditions with the melting temperature 60 °C. Samples were normalized to human β-actin (ACTB) as an endogenous reference. RT-qPCR was analyzed using the ΔΔC_T_ method [55]. The data were analyzed in Microsoft Excel and Prism 6.0.

### 4.10. Enzyme-Linked Immunosorbent Assay (ELISA)

Conditioned media was collected from cell lines treated with or without OpA (1 μM) for 6 h. The amount of human IL-8 protein in conditioned media was measured using human IL-8 ELISA kits according to the manufacturer’s protocol (ELH-IL8-1, RayBiotech, Peachtree Corners, GA, USA).

### 4.11. Identification of Differentially Expressed Genes

Library preparation and RNA-seq were conducted on triplicate samples using the services of Novogene (Sacramento, CA, USA). We used Salmon [56] to quantify the expression of transcripts from FASTQ files. We indexed the reads using the GRCh38 human genome with the following options: –threads 8 –gcBias –validate Mappings. Quantifications were imported to DESeq2 (v1.32.0) using the tximeta (v1.10.0) package in R (v4.1.1) [57,58]. DESeq2 was used to identify differentially expressed genes between the control and treated condition.

### 4.12. Gene Ontology and Gene Set Enrichment Analysis

Gene ontology analysis was performed using functional annotations for genes in the GRCh38 assembly of the human genome. We used the clusterProfiler (v4.0.5) package [59] to quantify the overrepresentation of differentially expressed genes in all three Gene Ontology categories: Biological Process, Molecular Function, and Cellular Component. We also tested for the enrichment of differentially expressed genes in KEGG pathways. We corrected for multiple testing using the Benjamini–Hochberg procedure and considered significance at a q-value < 0.05. Revigo was used to generate a condensed set of GO terms by inputting GO terms with *p*-value < 10^−10^.

### 4.13. Tumor Growth

Female Scid/bg (CB17.Cg-PrkdcscidLystbg-J/Crl) mice (3–4 weeks old) weighing approximately 20 g were obtained from Charles River Laboratories (Wilmington, MA, USA) and acclimatized for 2–3 weeks. Mice were maintained under a 12 h light/dark cycle at a temperature of 20 °C to 22 °C. Food and water were available ad libitum. Mice were maintained in accordance with the Institutional Animal Care and Use Baylor University Committee procedures and guidelines. MDA-MB-231 cells were harvested, pelleted by centrifugation at 2000× *g* for 2 min, and resuspended in sterile serum-free medium supplemented with 30% to 50% Matrigel (BD Biosciences). Cells (2 × 10^6^ in 100 µL aliquots) were implanted into the left fourth mammary fat of 6–8-week-old mice and allowed to grow orthotopically until measurable by caliper. Then, vehicle or OpA (suspended in DMSO) or docetaxel (AC456262500, Thermo Scientific) was administered by intraperitoneal injection once a week for 6 weeks at 5 mg/kg OpA or 5 mg/kg docetaxel to randomly assigned animals. Tumor volume and body weight were recorded concurrently with injection protocol. The progression of tumors was monitored every alternative day. Each group contained 6 mice, predicted to generate 80% power for an alpha of 0.05. Humane endpoints for this study were established based on clinical observations including a 20% decrease in body weight from the baseline, ulceration, tumor size beyond 2 cm, visible signs of distress, dehydration, or indication of any pain or discomfort in the animals. All the mice in the experiment were euthanized by CO_2_ inhalation, and death was verified by a lack of respiration and heartbeat.

### 4.14. Statistical Analyses

The experimental data were analyzed and plotted using GraphPad Prism 6.0. The mean between two groups were compared by Student’s *t* test, and the mean between multiple groups were compared by one-way ANOVA with Dunnet’s correction for multiple hypothesis testing. A *p*-value less than 0.05 was considered to be a significant difference. All data were obtained from at least three independent experiments.

## 5. Conclusions

In summary, our study suggests a novel mechanism by which OpA induces lytic cell death in breast cancer cells. We find that OpA triggers cellular swelling, membrane rupture, and cytokine release and is capable of inducing necrosomes in RIPK3-abundant cells. Targeting a potential RIPK1-GSDMD pathway could offer new therapeutic strategies for conditions characterized by excessive cytokine release such as sepsis, auto-immune disease, or inflammatory cancers [60,61]. The limitations of the study include a reliance on in vitro culture and the potential off-target effects of small-molecule inhibitors employed. Moreover, the complexity of cell death pathways leave open the possibility for a greater degree of crosstalk than anticipated. Nevertheless, these findings suggest the need for future investigation into the capacity of OpA to induce inflammatory cell death in vivo.

## Data Availability

RNA-seq data is publicly available via the Gene Expression Omnibus hosting by NIH at accession GSE277026. Other data will be made available upon reasonable request.

## Supplementary Materials

The following supporting information can be downloaded at: https://www.mdpi.com/article/10.3390/ijms27020618/s1.
