# Supplementary material for: Gasdermin D Cleavage and Cytokine Release, Indicative of Pyroptotic Cell Death, Induced by Ophiobolin A in Breast Cancer Cell Lines
Source: Int J Mol Sci. 2026 Jan 7;27(2):618. doi: 10.3390/ijms27020618 (PMC12841065; doi:10.3390/ijms27020618)
Supplement: Supplementary file 1 [file ijms-27-00618-s001.zip › ijms-3988737-supplementary.pdf]

Gasdermin D Cleavage and Cytokine Release, Indicative of Pyroptotic Cell Death, Induced by Ophiobolin A in Breast Cancer Cell Lines

Santhalakshmi Ranganathan [1], Tolulope Ojo [1], Alagu Subramanian [1], Jenna Tobin [1], Alexander Kornienko [2], Angela Boari [3], Antonio Evidente [4], Mary Lauren Benton [5], Daniel Romo [6] and Joseph H. Taube [1,*]

1 Department of Biology, Baylor University, Waco, TX 76706, USA;
2 Department of Chemistry and Biochemistry, Texas State University, San Marcos, TX 78666, USA
3 Institute of Sciences and Food Production, National Research Council, 70126 Bari, Italy
4 Institute of Biomolecular Sciences, National Research Council, 80078 Pozzuoli, Italy
5 Department of Computer Science, Baylor University, Waco, TX 76706, USA;
6 Department of Chemistry and Biochemistry, Baylor University, Waco, TX 76706, USA
* Correspondence: joseph_taube@baylor.edu

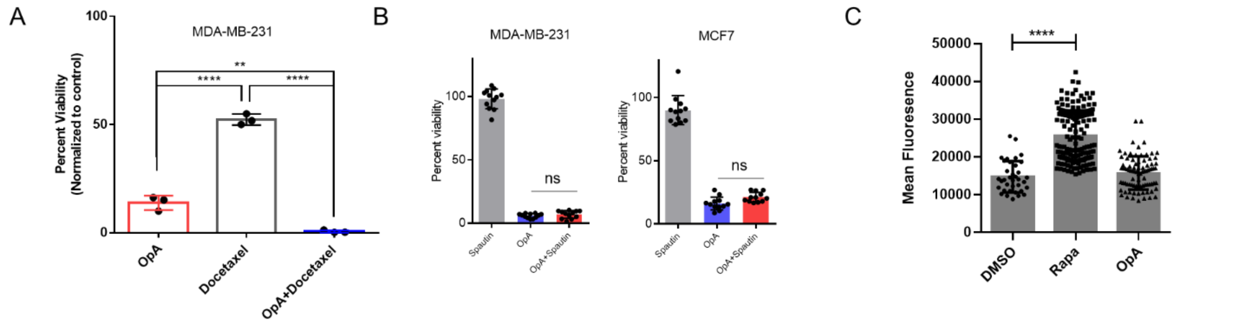

Figure S1. (A) Viability of MDA-MB-231 cells treated with OpA, docetaxel, or a combination for 24h. (B) Viability of OpA-treated cells in presence or absence of spautin-1 (1 μM) for 24h. The values presented are relative to the viability of untreated cells and normalized to 100%. (C) MDA-MB-231 cells were plated on glass-bottom slides for imaging, then treated with OpA or rapamycin for 24h before staining for autophagolysosomes using CytoID. Intensity of stain of individual cells in plotted. *p< 0.05, **p<0.01, ***p<0.001, ****p<0.0001, ns=not significant vs control using Student's t-test.

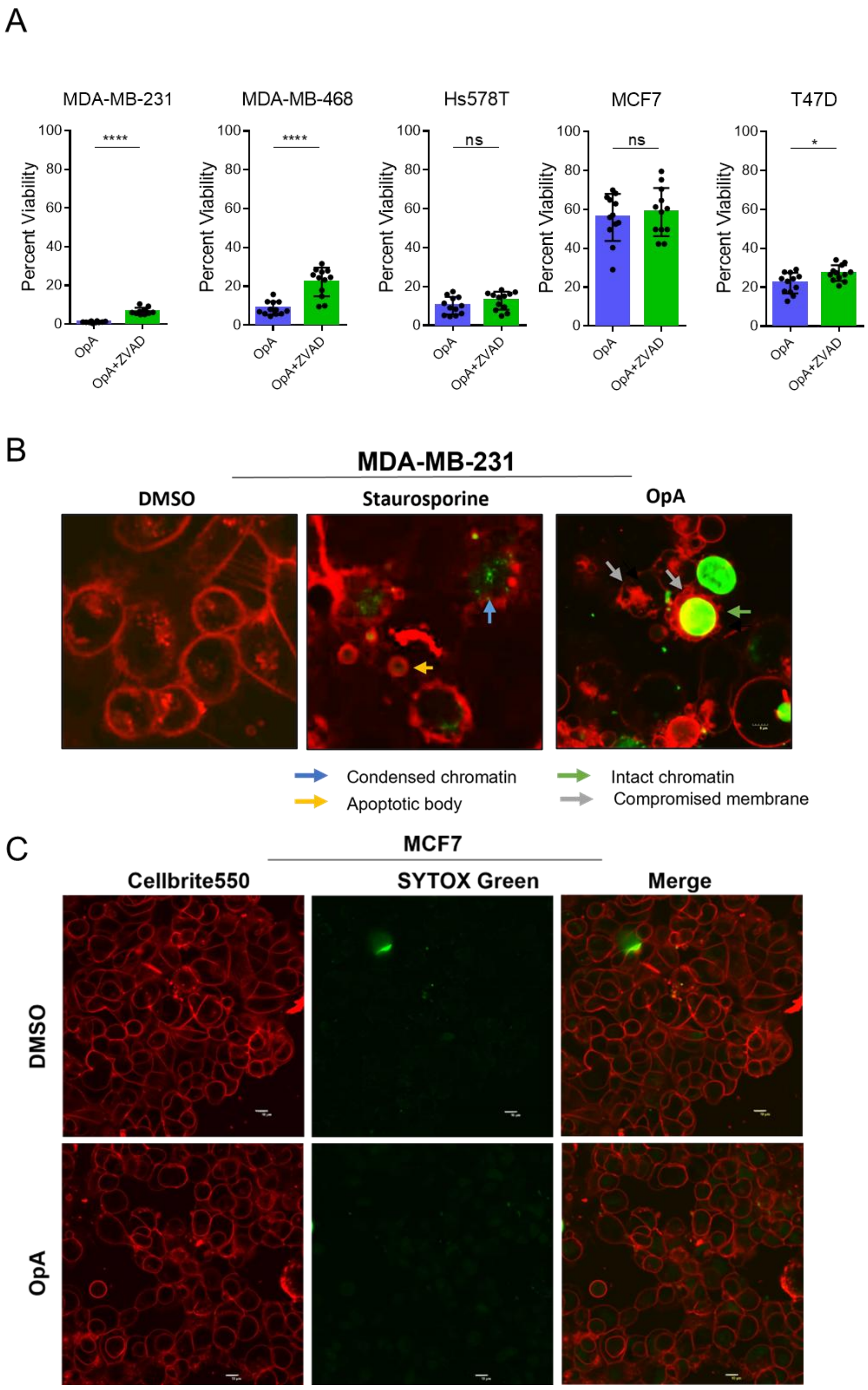

Figure S2. (A) Cell viability of 1 μM OpA-treated cells in presence or absence of 20 μM zVAD for 24h. The values presented are relative to the viability of untreated cells and normalized to 100%. (C) Confocal images showing apoptotic bodies and chromatin condensation in STS- but not OpA-treated MDA-MB-231 cells. (D) Confocal microscopy images of MCF7 cells showing plasma membrane stain (Cellbrite550) and lack of DNA stain (SYTOX green) in OpA (0.5 μM) treated versus control cells for 6h. Graphed data are presented as the mean ± SD from three independent experiments. *p< 0.05, **p<0.01, ***p<0.001, ****p<0.0001, ns=not significant vs control using Student's t-test.

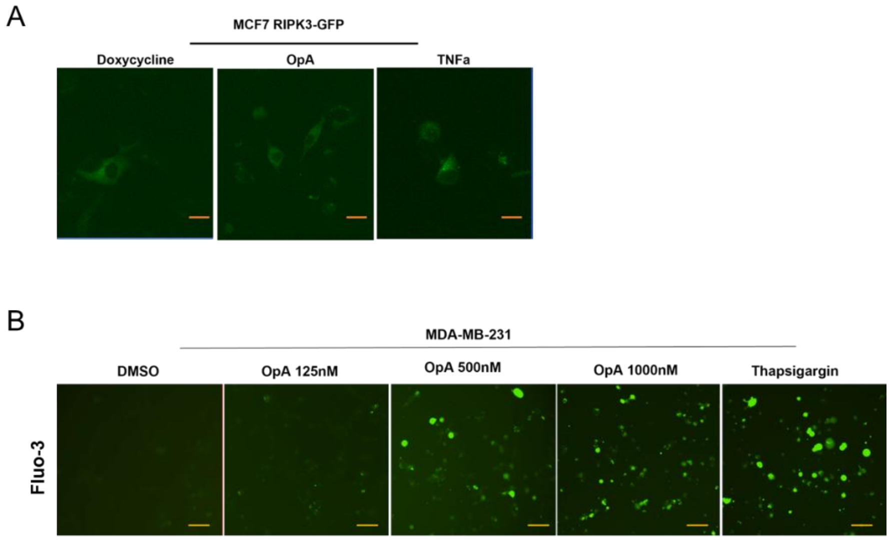

Figure S3. (A) Confocal images of MCF7 RIPK3-GFP cells showing GFP expression but no puncta upon OpA treatment for 24h. (B) Representative epi-fluorescence images of MDA-MB-231 cells showing calcium accumulation upon increasing dosage of OpA for 24h.
